# 345. Unaccounted Costs Associated with Different Outpatient Parenteral Antimicrobial Therapy (OPAT) Models of Care

**DOI:** 10.1093/ofid/ofad500.416

**Published:** 2023-11-27

**Authors:** Jacob Player, Rory Bouzigard, Mark Arnold, Norman S Mang, Brenton Hall, Michael Lane, Trish M Perl, Laila M Castellino

**Affiliations:** University of Texas Southwestern Medical School, Dallas, Texas; UT Southwestern, Dallas, Texas; University of Texas Southwestern Medical School, Dallas, Texas; Parkland Health, Dallas, Texas; Parkland Health and Hospital System, Dallas, TX; Parkland Health, Dallas, Texas; University of Texas Southwestern Medical Center, Dallas, Texas; University of Texas Southwestern Medical Center, Dallas, Texas

## Abstract

**Background:**

OPAT decreases length of stay/inpatient costs while benefiting patients. However, OPAT costs incurred in the ambulatory setting are poorly quantified. We evaluated unaccounted costs and potential savings from OPAT delivered via patient self-administration (S-OPAT), home care agencies/hemodialysis centers (HH-OPAT), and skilled nursing facilities (SNF-OPAT).

**Methods:**

The electronic health record (EHR) for all adult patients discharged on OPAT from Parkland Hospital (PH), a 900-bed safety-net hospital, during April - June 2021 and January - March 2022 was reviewed for the number and duration of antibiotics administered and post-discharge non-billable encounters (defined as an encounter on any day without a corresponding billable visit). Encounters on the day of a billable visit were excluded. Inpatient days avoided were defined as days post-discharge on which the patient received OPAT. An average daily hospital adjusted expense per inpatient day of $3,764 for Texas state hospitals and wholesale acquisition cost data from the Texas Department of State Health Services were used to estimate cost savings from OPAT. 340B pricing was used to calculate the cost of drugs provided to S-OPAT patients by PH. Antibiotics with different formulations were converted to equivalent daily doses and costs were averaged to estimate a daily antibiotic cost. The institutional IRB approved this study.

**Results:**

Of 340 patients, 84 (25%) received SNF-OPAT, 115 (34%) HH-OPAT, and 141 (41%) S-OPAT. There were 255 non-billable encounters in the S-OPAT group, 242 in the HH-OPAT, and 220 in the SNF-OPAT group, with the highest rate per 100 person-days in SNF-OPAT recipients (Table 1), nearly twice that of S-OPAT. Antibiotic costs avoided by discharging patients on OPAT were $383,240 for HH-OPAT patients and $505,588 for SNF-OPAT patients (Table 2). For S-OPAT patients, costs incurred by PH based on 340B pricing were $33,785.

**Figure:**
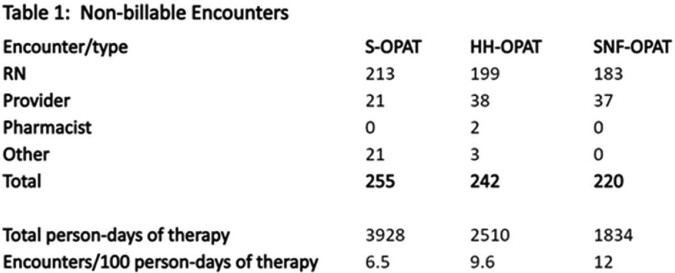

**Figure:**
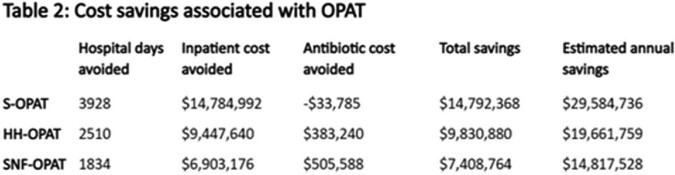

**Conclusion:**

All OPAT care models saved costs compared to hospitalization. Our study likely underestimates the unaccounted costs associated with OPAT; however, costs incurred may be significant and differ between care models. Personnel time and non-billable work should additionally be considered in determining true OPAT cost.

**Disclosures:**

**All Authors**: No reported disclosures

